# Successful eculizumab treatment of recurrent postpartum atypical hemolytic uremic syndrome after kidney transplantation 

**DOI:** 10.5414/CNCS108491

**Published:** 2015-06-15

**Authors:** Katherine Garlo, Doug Dressel, Marizela Savic, John Vella

**Affiliations:** 1 Department of Medicine,; 2 Department of Pathology, and; 3 Division of Nephrology & Transplantation, Maine Medical Center, Tufts University School of Medicine, Portland, ME, USA

**Keywords:** hemolytic uremic syndrome, eculizumab, complement, kidney transplant, postpartum

## Abstract

Postpartum atypical hemolytic uremic syndrome (aHUS) is a rare disorder associated with poor maternal and fetal outcomes. We describe a case of severe postpartum aHUS with recurrence in a kidney allograft after a second pregnancy. The patient had initially presented age 28 years with aHUS that developed after her first delivery. In spite of treatment with plasma exchange, she developed end-stage renal disease (ESRD) requiring years of hemodialysis before receiving a kidney transplant from a living unrelated donor. Two years later, she became pregnant again and at 26 weeks gestation she presented to our hospital with hypertension and proteinuria. Within 48 hours of delivery she developed hemolytic anemia, thrombocytopenia, and oliguric acute kidney injury (AKI) culminating in the need for dialysis. There was no response to therapeutic plasma exchange (TPE). However, treatment with eculizumab led to prompt, successful resolution of hemolysis, thrombocytopenia, and AKI. Three months after therapy was stopped, her disease relapsed causing renal failure again requiring dialysis. At that time, an allograft biopsy revealed severe thrombotic microangiopathy (TMA). Eculizumab was resumed without plasma exchange leading to resolution of aHUS and return of kidney function. Now, her baby is nearly 2 years old. She remains on maintenance eculizumab therapy 1,200 mg every 2 weeks without dialysis. She has excellent renal function with creatinine of 1.2 mg/dL, eGFR 52 mL/min/1.73 m, and proteinuria 0.35 g/day. She will likely be on eculizumab for the remainder of her life.

## Introduction 

Atypical hemolytic uremic syndrome (aHUS) is a rare disorder characterized by dysregulation of the alternative pathway of the complement system that accounts for 5 – 10% of all cases of HUS. Contrary to typical HUS, there is no association with enterocytopathic-shigatoxin producing bacteria (e.g., *Escherichia coli* 0157:H7) that causes hemorrhagic diarrhea most commonly in children [1]. Approximately 50% of aHUS cases have been linked to genetic disorders, 5% to autoimmune disease while the remainder is incompletely understood. Most of the genetic mutations associated with this condition lead to loss of function of inhibitory proteins of the complement alternative pathway resulting in uncontrolled amplification [2]. Complement, as part of the innate immune system, triggers release of anaphylatoxins leading to opsonization, and direct cell lysis via the membrane attach complex. The alternative pathway is highly regulated by inhibitory proteins that allow for controlled continuous activity in preparation for exposure to foreign antigen but prevent inappropriate amplification. Gene mutations and polymorphisms in the alternative complement pathway, as well as autoantibodies to regulatory proteins of the alternative complement pathway, have been identified in patients with sporadic and familial aHUS. Familial cases have been shown to be associated with increased prevalence of gene mutations and severe disease. Certain gene mutations have been linked with poor prognostic factors including earlier onset of disease, higher mortality, poor response to treatment, and relapse after kidney transplant [2]. 

Pregnancy has been identified as a trigger for aHUS with a reported incidence of 1 : 25,000 gestations. The majority of cases present postpartum within 48 hours, although it can develop as early as the second trimester. Postpartum aHUS is associated with poor outcomes leading to maternal mortality in ~ 25% of cases, pre-eclampsia in 18% of cases, and fetal loss occurs in 5% of the cases [3]. Nearly 60% of maternal survivors develop end-stage renal disease (ESRD) requiring long-term dialysis or kidney transplantation. Recurrent disease after transplantation affects half of patients and generally leads to graft failure [4]. 

Conventional management of aHUS includes therapeutic plasma exchange (TPE) and hemodialysis [5]. Unfortunately, this strategy is ineffective in many patients with severe or recurrent disease after kidney transplantation. Eculizumab is a novel targeted therapy that controls over-amplification of complement through inhibition of complement factor 5. It was approved by the Federal Drug Administration for treatment of aHUS in 2012 and is currently being investigated for use in a variety of other diseases linked to impaired regulation of the complement system [6]. 

## Case presentation 

The patient had initially presented to another institution age 28 years with acute kidney injury (AKI), thrombocytopenia, and hemolytic anemia within days after delivering her first baby. She had undergone two first trimester spontaneous abortions although there was no past or family history of clotting disorders, thromboembolism, autoimmune disease, or recreational drug use. She was diagnosed with thrombotic microangiopathy and treated with plasma exchange for and hemodialysis for AKI that led to end-stage renal disease (ESRD). Testing for systemic lupus erythematic (SLE) and anti phospholipid antibody syndrome (APLAS) were negative. 

She underwent 2 years of hemodialysis before receiving a living donor renal transplant from her husband in 2010. Transplantation was complicated by a BANFF 1b acute rejection within the first year, which was successfully treated with rabbit anti thymoglobulin (rATG). She was maintained on three-drug immunosuppression with low dose prednisone, tacrolimus, and mycophenolate mofetil. Until her second AKI presentation, she had benefited from excellent graft function without further rejection or infection (creatinine of 0.8 mg/dL, eGFR 96 mL/min/1.73 m^2^). She had been advised not to become pregnant again due to risk for thrombotic microangiopathy (TMA) and further kidney injury. However, she remained sexually active, and attempts to pursue appropriate birth control were unsuccessful. 

**Figure 1.**
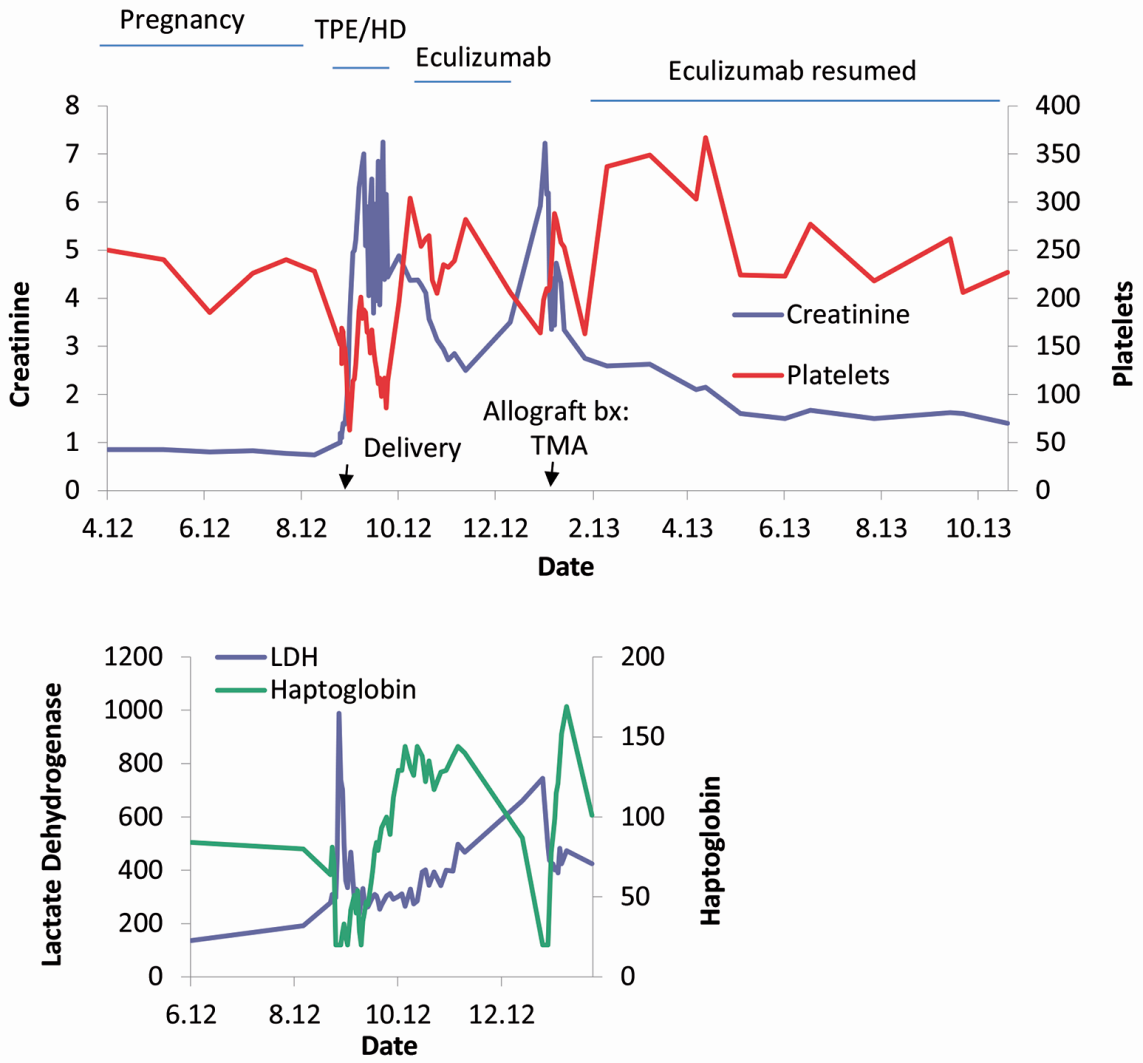
Clinical course of recurrent postpartum aHUS in a kidney allograft: response to eculizumab. The patient had a normal pregnancy until 26 weeks gestation when she was hospitalized for hypertension and proteinuria. After caesarian delivery for pre-eclampsia, the patient developed thrombocytopenia, hemolytic anemia, and AKI consistent with aHUS. Her hospital course and outpatient follow-up demonstrate successful response to eculizumab with relapse of disease once therapy was withdrawn and full recovery after therapy was reinstated. Her laboratory studies show return to baseline creatinine, resolution of thrombocytopenia, and cessation of hemolysis with normalization haptoglobin and lactate dehydrogenase with eculizumab. The dates of hospitalization were 09/12/2012 – 10/12/2012.

Two years post transplantation, she presented to our institution at age 32 years and pregnant at 26 weeks gestation with hypertension and proteinuria. Her creatinine was 0.8 mg/dL. She underwent emergency caesarian section for pre-eclampsia. Within 48 hours of delivery she developed thrombocytopenia, hemolytic anemia, and AKI (Figure 1). Pertinent physical findings included BP 160/94, a grade 2/6 mid systolic murmur at the right upper sternal border, bilateral pulmonary rhales, and 3+ pitting edema. Her urine output was 200 – 400 mL/day. Pertinent laboratory values revealed anemia with evidence of hemolysis and thrombocytopenia (Table 1). Creatinine rose to 7.0 mg/dL, eGFR 8 mL/min/1.73 m^2^. All other blood testing, including liver enzymes were within normal limits. Urinalysis was notable for 500 mg/dL protein and 50 RBCs per high power field. Urine spot protein to creatinine ratio was 10.8. A disintegrin and metalloproteinase with a thrombospondin type 1 motif, member 13 (ADAMTS13) activity level was within normal limits at 75%. Testing for autoimmune diseases and hypercoaguability were negative. Hydralazine was stopped and belatacept was substituted for tacrolimus for their potential causative role in aHUS. She was initially treated traditionally with nine courses of plasma exchange and hemodialysis without clinical improvement. Prior to starting eculizumab, she was vaccinated for *Neisseria meningitides* and *Streptococcus pneumonia* due to the increased risk of infection from impaired opsonization and phagocytation of encapsulated bacteria. She also received vaccination for the annual strains of influenza and tetanus diphtheria acellular pertussis, antibody levels were not checked and she was started on ciprofloxacin 500 mg daily for prophylaxis. She received 4 weeks of eculizumab 900 mg infusion followed by 1,200 mg infusion on the 5^th^ week. She also received 300 mg supplemental dosing post plasma exchange. Within 2 weeks of starting eculizumab therapy, there was complete successful resolution of thrombocytopenia, hemolysis, and renal failure with return to baseline kidney function (Figure 1). 

**Figure 2.**
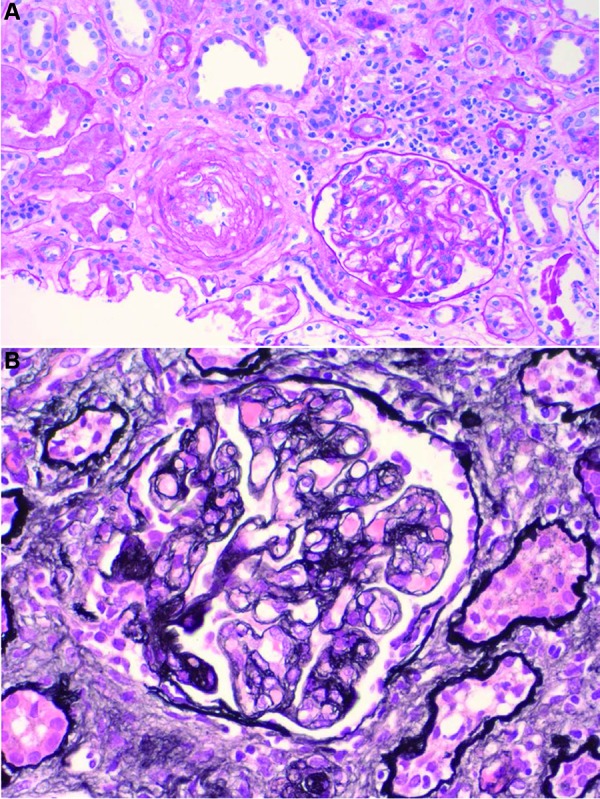
Light microscopy of kidney biopsy after withdrawal of eculizumab. a: Glomerulus with segmental membranoproliferative glomerulonephritis (MPGN) pattern and multiple large intravascular fibrin thrombi, vessel with thickened media with onion skinning pattern, findings are consistent with TMA. PAS stain 20×. b: A glomerulus with multiple intracapillary fibrin thrombi, MPGN pattern, basement membrane reduplication, and cellular interposition. Vessel showing thickened media. Jones BM stain 40×.

Approximately 2 months after eculizumab was withdrawn, she developed full relapse of aHUS with recurrence of thrombocytopenia, hemolysis, and AKI once again requiring dialysis. Kidney biopsy of the allograft at the time showed severe TMA without evidence of graft rejection (Figure 2). Eculizumab was restarted with 1,200 mg every 2 weeks without plasma exchange. Now, nearly 1 year later, she remains on this dose of eculizumab 1,200 mg every 2 weeks as maintenance therapy with the anticipation to continue life-long. The cost of the medication is $73,043.52 for the induction period and $24,347.84 per maintenance dose leading to a maintenance cost of $633,043.84 per year. She is also on immunosuppression with mycophenolate mofetil (MMF ) 360 mg twice daily, prednisone 5 mg daily, belatacept 450 mg monthly, and ciprofloxacin 500 mg daily as bacterial infection prophylaxis. She has excellent graft function with creatinine of 1.2 mg/dL, eGFR 52 mL/min/1.73 m, and proteinuria 0.35 g/day. She has been evaluated for mutations of regulatory proteins as well as testing for functional and quantitative enzyme deficiencies in the complement system (Table 2). 

## Discussion 

The diagnosis of aHUS is clinical, based on symptoms and laboratory testing. Classically, symptoms relate to hemolysis, thrombocytopenia, and AKI. Our patient clearly had evidence of intravascular hemolysis, AKI, and subsequently underwent kidney allograft biopsy confirmation of MTA. Diagnostic testing to identify the cause of aHUS is challenging. Genetic studies, enzyme assays identifying level and function, and autoantibody serologies are available options for testing. To date, nine genes involved in regulation of the complement pathway have been identified. The most common genetic mutations are complement factor H (CFH), complement factor I (CFI), and membrane cofactor protein (MCP) also known as CD46. These mutations are inherited autosomal dominant or recessive and have incomplete penetrance (Table 2) [2, 4]. Up to 80% of the cases of postpartum aHUS have genetic abnormalities of the alternative complement pathway. These tests have prognostic value; mutations are associated with increased risk of relapse and progression to ESRD. In 2012, a case of postpartum aHUS was reported in a young woman with a liver transplant. Genetic studies of her liver donor showed a nonsense CFH mutation leading to factor H deficiency. The patient’s genotype showed that she was homozygous for MCP (CD46). Her disease was attributed to an acquired CFH mutation from the donor in conjunction with a common susceptibility factor MCP (CD46) haplotype in the patient triggered by pregnancy. She developed ESRD requiring dialysis and was never treated with eculizumab [7]. A second case report in 2014 of a patient with genetic aHUS from homozygous CFH mutation developed relapse of aHUS after pregnancy. This patient’s disease also failed to respond to plasma exchange however, unlike the previous case, she was successfully treated with eculizumab to achieve complete clinical remission [8]. Despite detailed testing, we were unable to identify the cause of our patient’s aHUS. The levels of C3 and CFH in our patient were borderline low and likely not severely depressed enough to cause clinical disease. She was referred to a genetic counselor for discussion of genetic testing and risk for her children but was not able to follow-up for financial reasons. 

Eculizumab is a humanized monoclonal antibody to complement factor 5 that was FDA approved for paroxysmal nocturnal hemoglobinuria in 2007 and for pediatric aHUS in 2011. Its use is being expanded to a wider patient population and a diversity of diseases. A randomized controlled clinical trial of eculizumab in 37 adults with aHUS showed resolution of TMA, thrombocytopenia, and return of kidney function [9]. As supported by this case report, eculizumab appears to be especially effective in recurrent TMA post-transplant [5, 10, 11]. Treatment with C5 inhibition may also be efficacious in patients with aHUS who have received a long course of dialysis prior to eculizumab treatment [12]. A case report of a patient with CFH mutation developed postpartum aHUS within 7 days of delivery. Her disease failed to respond to plasma exchange requiring dialysis. She achieved successful remission with eculizumab and suffered relapse of disease after discontinuing eculizumab requiring long-term maintenance therapy [13]. Our patient had a similar course with relapse of aHUS after withdrawal of eculizumab. She receives maintenance eculizumab therapy 1,200 mg every 2 weeks and is expected to continue for the remainder of her life. Though the cost is expensive, $24,347.84 per maintenance dose leading to a total maintenance cost of $633,043.84 per year, eculizumab has regulated her alternative complement pathway preventing further relapse of disease and providing stable allograft function off dialysis. 

There is little known about the long-term safety and efficacy of terminal complement inhibition. A study of 195 patients with PNH from the initial phase II and III trials for eculizumab were enrolled in an open-label extension study. They received standard initiation dosing of eculizumab followed by maintenance dosing of 900 mg every 14 days and were followed for 66 months. There were 4 patient deaths, which were not attributed to eculizumab, yielding 97.6% survival. All participants had complete resolution of thrombocytopenia and hemolysis. They also exhibited stabilization or improvement of chronic kidney disease (CKD). The authors report that the treatment was well tolerated with no evidence of cumulative toxicity [14]. A meta-analysis of the long-term efficacy of eculizumab in aHUS that included two uncontrolled prospective studies with a total of 37 patients (adults and children) showed that TMA event-free status was achieved in 84% of patients and sustained longer than 26 weeks [6]. The most frequent adverse events were upper respiratory tract infections in nearly 33% of the patients and there were no deaths or cases of meningococcal septicemia. 

This clinical report demonstrates a unique case of recurrent postpartum aHUS in a kidney allograft. The patient failed to respond to traditional therapeutic plasma exchange and benefited from complete resolution of disease with eculizumab. Upon withdrawing eculizumab, she suffered disease relapse as proven with biopsy of the kidney allograft showing severe TMA. She is now on lifelong maintenance eculizumab with stable kidney function, no dialysis, and healthy children. Postpartum aHUS is rare and has historically been associated with a very poor maternal and fetal prognosis. The story of our patient highlights the success of targeted therapy to control the alternative complement pathway in aHUS. 

## Acknowledgments 

There are no sources of funding to acknowledge; however, we would like to thank all our colleagues in nephrology and primary care who have helped care for this patient. 

## Conflict of interest 

Dr. Vella receives research funding from Bristol Myers Squib and Astellas. We have no other conflicts of interest. 

**Table 1.** Pertinent initial laboratory test results.

Lab test	Patient’s result (normal range)
Hemoglobin	6.5 (11.8 – 15.8) g/dL
MCV	96 (80 – 100) fL
Platelets	89 (140 – 440) Thou/uL
AST	35 (0 – 37) U/L
ALT	19 (0 – 40) U/L
ALK PHOS	70 (39 – 117) U/L
LDH	975 (94 – 250) U/L
Haptoglobin	< 20 (25 – 200) mg/dL
Blood smear	2 + schistocytes (none expected)
ADAMTS13	75% (50 – 160%)

MCV = mean corpuscular volume; AST = liver enzymes aspartate aminotransferase; ALT = alanine aminotransferase; ALK PHOS = alkaline phosphatase; LDH = lactate dehydrogenase. A disintegrin and metalloproteinase with a thrombospondin type 1 motif, member 13 (ADAMTS13) activity level was measured with a direct activity assay using Fluorescence Resonance Energy Transfer [FRETS] with the VWF (Von Willebrand Factor) 73 fragment as the substrate. Severe deficiency as in TTP is 5 – 10% activity level.

**Table 2.** Complement pathway proteins involved in the pathogenesis of TMA.

Mutation	Estimated prevalence	Estimated penetrance	Case patient (normal range)	Proposed function
CFH	30%	48%	Level: 183 (60 – 412) ncg/mL	Accelerates C3 decay, cofactor for CFI
CD46	12%	53%	Not measured	Membrane cofactor Protein cofactor
CFI	5 – 10%	50%	Level: 38.2 (29.3 – 58.5) ncg/mL	Degrades C3 convertase
C3	5%	56%	Level: 559 (0 – 780) ng/ml Function: 213 (11,249 – 42,887) U/mL*	Anaphylatoxin and opsonin
CFB	5%	64%	Level: 197.3 (127.6 – 278.5) ncg/mL Function: 59 (123 – 426) U/mL** CFB C1 Esterase inhibitory autoantibody IgG 4.2 (0.89 – 36.1)%	Cleaves C3/C5 protein complex
C4	Unknown	Unknown	Level 19.3 (19 – 52) mg/dL Function: 1,437,302 (400,000 – 430,000,000) U/mL	Anaphylatoxin and opsonin
C1	Unknown	Unknown	Function: 144 (74 – 174)%	Initiation of the classical complement pathway
C1 autoantibody	Unknown	Unknown	Function: 0.3 (0.0 – 7.0)%	

Adopted from [2]. *C3 function low 213 verified by repeated analysis ref range 11,249 – 42,887 U/mL. **Factor B function low 59 U/mL verified by repeated analysis 123 – 426 U/mL. All testing was done at National Jewish Health in Denver, CO, USA.
